# Relationship between non-invasively detected liver fibrosis and in-hospital outcomes in patients with acute coronary syndrome undergoing PCI

**DOI:** 10.1007/s00392-022-02078-z

**Published:** 2022-08-11

**Authors:** Flavio Giuseppe Biccirè, Francesco Barillà, Emanuele Sammartini, Edoardo Maria Dacierno, Gaetano Tanzilli, Daniele Pastori

**Affiliations:** 1 Department of General and Specialized Surgery “Paride Stefanini, Sapienza University of Rome, Rome, Italy; 2 Department of Systems Medicine, University of Rome Tor Vergata, Rome, Italy; 3 Department of Clinical Internal, Anesthesiologic and Cardiovascular Sciences, Sapienza University of Rome, Viale del Policlinico 155, 00161 Rome, Italy

**Keywords:** Acute coronary syndrome, Liver fibrosis, Myocardial infarction, Cardiogenic shock, Adverse events

## Abstract

**Background:**

Patients with acute coronary syndrome (ACS) undergoing percutaneous coronary intervention (PCI) still experience a high rate of in-hospital complications. Liver fibrosis (LF) is a risk factor for mortality in the general population. We investigated whether the presence of LF detected by the validated fibrosis 4 (FIB-4) score may indicate ACS patients at higher risk of poor outcome.

**Methods:**

In the prospective ongoing REAl-world observationaL rEgistry of Acute Coronary Syndrome (REALE-ACS), LF was defined by a FIB-4 score > 3.25. We repeated the analysis using an APRI score > 0.7. The primary endpoint was in-hospital adverse events (AEs) including a composite of in-hospital cardiogenic shock, PEA/asystole, acute pulmonary edema and death.

**Results:**

A total of 469 consecutive ACS consecutive patients were enrolled. Overall, 21.1% of patients had a FIB-4 score > 3.25. Patients with LF were older, less frequently on P2Y12 inhibitors (*p* = 0.021) and admitted with higher serum levels of white blood cells (*p* < 0.001), neutrophils to lymphocytes ratio (*p* < 0.001), C-reactive protein (*p* = 0.013), hs-TnT (*p* < 0.001), creatine-kinase MB (*p* < 0.001), D-Dimer levels (*p* < 0.001). STEMI presentation and higher Killip class/GRACE score were more common in the LF group (*p* < 0.001). 71 patients experienced 110 AEs. At the multivariate analysis including clinical and laboratory risk factors, FIB-4 > 3.25 (OR 3.1, 95%CI 1.4–6.9), admission left ventricular ejection fraction% below median (OR 9.2, 95%CI 3.9–21.7) and Killip class ≥ II (OR 6.3, 95%CI 2.2–18.4) were the strongest independent predictors of in-hospital AEs. Similar results were obtained using the APRI score.

**Conclusion:**

LF detected by FIB-4 score > 3.25 was associated with more severe ACS presentation and worse in-hospital AEs irrespective of clinical and laboratory variables.

**Graphical abstract:**

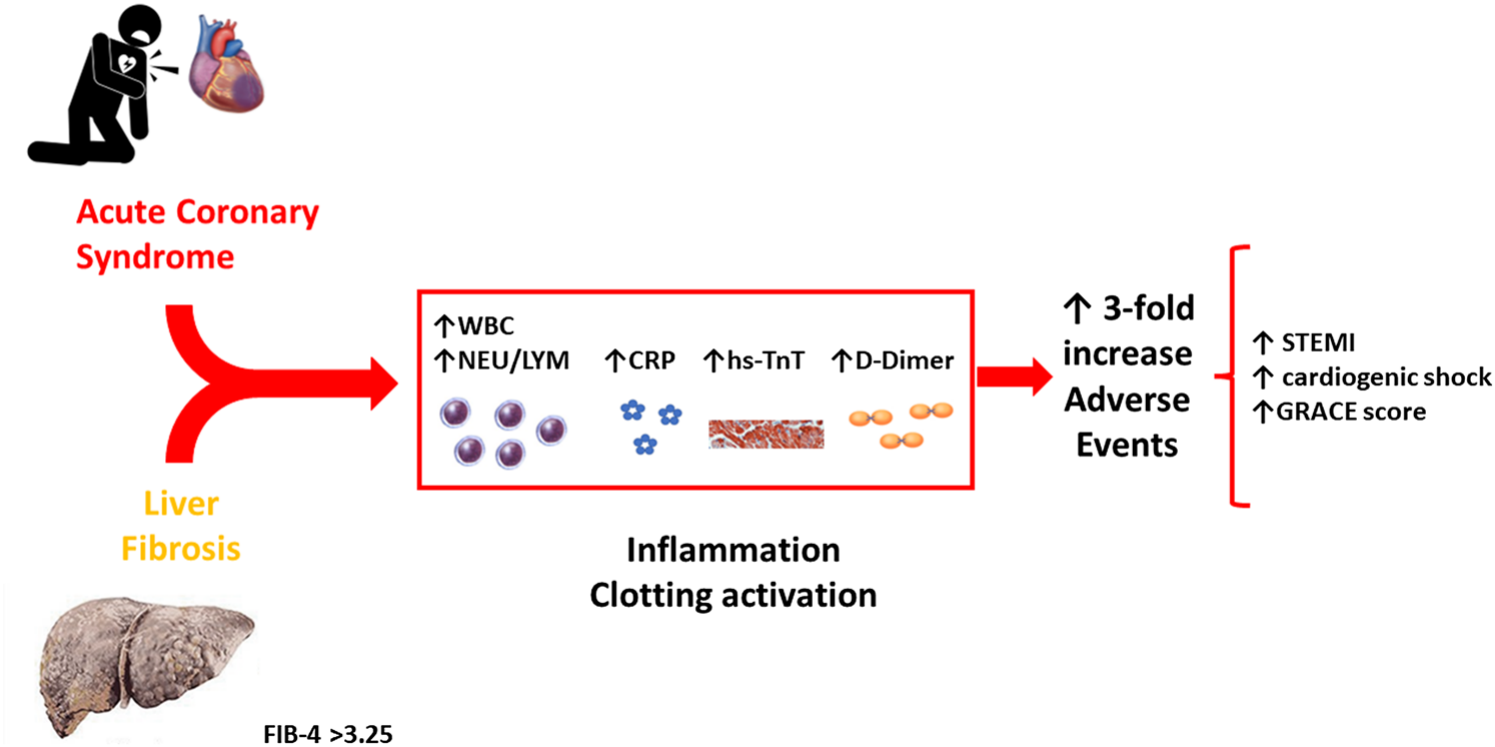

**Supplementary Information:**

The online version contains supplementary material available at 10.1007/s00392-022-02078-z.

## Introduction

Patients with acute coronary syndrome (ACS) still suffer from high in-hospital morbidity/mortality and long-term poor prognosis, including recurrence of myocardial infarction (MI) in about 10% of cases [1–3], despite advances in revascularization procedures techniques and current best medical therapy. This residual high cardiovascular risk may be driven by several factors including poor adherence to prescription [4], not reaching therapeutic target for comorbidities, or by the presence of non-cardiovascular risk factors [5], such as chronic kidney and liver disease.

In addition, a proportion of patients may experience early complications after ACS, including cardiogenic shock, arrhythmia, and in-hospital death. However, the clinical and biochemical characteristics of patients at risk of early complications after ACS are not well established, and so far, the use of common biomarkers of disease severity, such as high-sensitive troponin, did not lead to an improvement in clinical management of these patients [6].

In the past decades, given the impossibility of performing liver biopsies on large scale, different non-invasive biochemical markers have been proposed to identify patients with advanced liver damage. Among others, the AST-to-platelet ratio index (APRI) and the Fibrosis 4 (FIB-4) score, have been shown to be reliable tools for predicting liver fibrosis (LF) [7, 8].

Previous evidence also showed that a high FIB-4 was associated with an increased rate of long-term cardiovascular events in patients with cardiovascular disease, such as those affected by atrial fibrillation (AF) [9]. In addition, recent studies highlighted the association of LF with the severity of coronary artery disease (CAD) and advanced high-risk coronary plaque [10, 11].

Nevertheless, the impact of LF on in-hospital and short-term complications in patients admitted with ACS undergoing percutaneous coronary intervention (PCI) has been poorly investigated.

Our aim was to study the characteristics of patients hospitalized for ACS presenting with high FIB-4, and to investigate the relationship between LF and in-hospital outcomes.

## Methods

The REAl-world observationaL rEgistry of Acute Coronary Syndrome (REALE-ACS) is an ongoing multicentric registry collecting data on characteristics, management and outcomes of consecutive patients admitted for ACS at the Department of Clinical Internal, Anesthesiologic, and Cardiovascular Sciences, Sapienza University of Rome, Rome, Italy and Emergency Medicine Unit Department (from January 2016).

Patients with age < 18 years were excluded. Diagnosis of non-ST-elevation ACS (i.e., unstable angina [UA] and non-ST-elevation myocardial infarction [NSTEMI]) and ST-elevation myocardial infarction (STEMI) were made according to the latest European Society of Cardiology guidelines [6, 12].

At baseline, demographic characteristics and clinical information of each study patient were recorded as following: age, sex, anthropometric data, cardiovascular risk factors (hypertension, diabetes mellitus, smoking (former + current), hypertriglyceridemia, hypercholesterolemia), previous history of CAD, heart failure, ischemic stroke, peripheral artery disease and Global Registry of Acute Coronary Events (GRACE) score. Standard 12-lead ECGs were collected at admission and during in-hospital stay.

### Blood parameters

On admission, levels of aspartate aminotransferase AST/GOT (U/L), alanine aminotransferase ALT/GPT (U/L), C-reactive protein (CRP, upper limit of normal [ULN] < 0.5 mg/dL), hemoglobin (g/dL; anemia women < 12 g/dL and men < 13 g/dL), platelets × 10^9^/L, white blood cells (WBCs) × 1000, neutrophils (%), lymphocytes (%), neutrophils/lymphocytes ratio, D-Dimer (ULN < 450 ng/mL), glycemia (mg/dL), creatinine (mg/dL), and high-sensitive troponin T (hs-TnT, ULN < 0.014 μg/L) were collected. Thrombocytopenia was defined by a platelet count < 150 × 10^9^/L. Low serum albumin was defined as < 36 mg/L. Estimated glomerular filtration rates (eGFR) were determined using MDRD formula.

### Definition of liver fibrosis

LF was defined by a FIB-4 score > 3.25, a validated non-invasive score determined by applying the following formula: (age [years] × AST [U/L])/(platelet count [10^9^/L] × √ ALT [U/L]) [7, 8, 13]. We also used another non-invasive validated score to predict LF, namely APRI score by applying the following formula: (AST [U/L]/platelet count [10^9^/L]) [7, 8, 13].

### Clinical outcomes

The primary endpoint was a composite of in-hospital adverse events (AEs) including cardiogenic shock, pulseless electrical activity (PEA)/asystole, acute pulmonary edema and death. For the analysis, only the first event occurrence was considered.

The study was performed according to the Declaration of Helsinki.

### Statistical analysis

Categorical variables were reported as counts and percentage. Continuous variables were expressed as mean and standard deviation and compared by Student *t* test. *χ*^2^ test was used to compare proportions. A first descriptive analysis according to presence of high FIB-4 was performed. We also described clinical and biochemical characteristics associated with in-hospital AEs. A multivariable logistic regression analysis was used to calculate the relative odds ratio (OR) and 95% confidence interval (95%CI) for each factor associated with in-hospital AEs.

In the multivariable model, only clinical variables with a *p* value < 0.100 at univariable analysis were included. In this model, we tested for collinearity to avoid an over adjustment of the model given the limited number of events. The variables entered in the multivariable model were FIB-4 > 3.25, below median of left ventricular ejection fraction (LVEF) value obtained for entry echocardiography, Killip Class ≥ 2, MDRD < 49 mL/min (value obtained from receiver operating characteristic [ROC] curve analysis to optimize the cut-off). We also performed a subgroup analysis on patients with albumin, CRP, and D-Dimer available values.

Only *p* values < 0.05 were considered as statistically significant. All tests were two tailed and analyses were performed using computer software packages (SPSS-25, SPSS Inc. and MedCalc).

## Results

### Clinical and laboratory findings

A total of 469 consecutive ACS patients were included. Among them, 12 patients had a history of chronic liver disease (6 patients viral hepatitis, 4 alcoholic disorder and 2 both). Clinical characteristics of patients with or without a high FIB-4 score are summarized in Table 1. Overall, 21.1% of patients had a high FIB-4 score. Patients with LF were older (*p* < 0.001), less frequently overweight (*p* = 0.026) and more frequently affected by permanent AF (*p* = 0.033).

**Table 1:** Characteristics of ACS patients with FIB-4 above or below/equal to 3.25

	Overall (*n* = 469)	FIB-4 ≤ 3.25 (*n* = 305)	FIB-4 > 3.25 (*n* = 164)	*p*
Age (years)	65.7 ± 13.0	64.6 ± 12.2	69.9 ± 14.9	< 0.001
Women (%)	108 (23.0)	86 (23.2)	22 (22.2)	0.894
Body mass index (kg/m^2^)	26.7 ± 4.3	26.9 ± 4.4	25.8 ± 4.1	0.026
Risk factors				
Hypertension (%)	377 (80.4)	300 (81.1)	77 (77.8)	0.477
Diabetes (%)	142 (30.3)	111 (30.0)	31 (31.3)	0.806
COPD (%)	59 (12.6)	46 (12.4)	13 (13.1)	0.865
Smoking habit (%)	332 (71.4)	269 (73.3)	63 (64.3)	0.101
Current smoking (%)	187 (40.2)	150 (40.9)	37 (37.8)	0.643
PAD (upper + lower) (%)	95 (20.3)	71 (19.2)	24 (24.2)	0.263
Congestive heart failure history (%)	36 (7.7)	31 (8.4)	5 (5.1)	0.395
Thyroid disease (%)	42 (9.0)	38 (10.3)	4 (4.0)	0.072
History of stroke or TIA (%)	27 (5.8)	21 (5.7)	6 (6.1)	0.812
History of cancer (%)	58 (12.4)	45 (12.2)	13 (13.1)	0.864
Prior MI (%)	130 (27.8)	108 (29.3)	22 (22.4)	0.206
Home therapy				
ACE inhibitors (%)	241 (53.5)	171 (54.1)	43 (51.2)	0.712
Aldosterone receptor antagonists (%)	11 (2.8)	8 (2.5)	3 (3.6)	0.706
Beta blockers (%)	139 (34.8)	113 (35.8)	26 (31.0)	0.442
Acetylsalicylic acid (%)	167 (41.8)	134 (42.4)	33 (39.3)	0.621
P2Y12 inhibitors (%)	68 (17.0)	61 (19.3)	7 (8.3)	0.021
DAPT (%)	49 (12.3)	44 (13.9)	5 (6.0)	0.060
Acetylsalicylic acid or P2Y12 inhibitors (%)	186 (46.5)	151 (47.8)	35 (41.7)	0.328
Proton pump inhibitors (%)	138 (34.5)	113 (35.8)	25 (29.8)	0.366
Oral anticoagulants (any) (%)	28 (7.0)	25 (7.9)	3 (3.6)	0.229
Non-dihydropyridine calcium channel blockers (%)	9 (2.3)	8 (2.5)	1 (1.2)	0.692
Dihydropyridine calcium channel blockers (%)	106 (26.5)	85 (26.9)	21 (25.0)	0.782
Ranolazine (%)	23 (5.8)	19 (6.0)	4 (4.8)	0.797
Statins (%)	124 (31.0)	105 (33.2)	19 (22.6)	0.064
Cholesterol-absorption inhibitors (%)	9 (2.3)	6 (1.9)	3 (3.6)	0.404
Statins/cholesterol-absorption inhibitors association (%)	9 (2.3)	9 (2.8)	0 (0.0)	0.214
Fibrates (%)	3 (0.8)	3 (0.9)	0 (0.0)	1.000
Oral antidiabetic drugs (%)	76 (19.0)	57 (18.0)	19 (22.6)	0.350
Insulin therapy (%)	28 (7.0)	23 (7.3)	5 (6.0)	0.812
Uric acid lowering therapy (%)	19 (4.8)	15 (4.7)	4 (4.8)	1.000
Laboratory data				
Creatinine (mg/dL)	1.09 ± 0.88	1.09 ± 0.94	1.09 ± 0.59	0.982
eGFR MDRD (mL/min)	82.6 ± 30.6	83.3 ± 30.5	79.9 ± 30.8	0.326
eGFR < 60 mL/min (%)	93 (19.8)	69 (18.6)	24 (24.2)	0.256
Albumin (g/L) (*n* = 417)	39.4 ± 4.8	39.69 ± 4.79	38.31 ± 4.88	0.018
Albumin < 36 g/L (%) (*n* = 417)	76 (18.2)	54 (16.4)	22 (25.3)	0.062
C-reactive protein (mg/dL) (*n* = 356)	0.6[0.2–1.6]	0.6[0.2–1.3]	1[0.3–3.1]	0.013
Hemoglobin (g/dL)	14.4 ± 6.9	14.50 ± 7.71	13.99 ± 2.13	0.515
Anemia (%)	93 (19.8)	68 (18.4)	25 (25.3)	0.155
Platelets (× 10^9^/L)	243.1 ± 80.2	247.38 ± 79.44	227.08 ± 81.37	0.025
Thrombocytopenia < 150 × 10^9^/L (%)	26 (5.5)	16 (4.3)	10 (10.1)	0.044
AST/GOT (U/L)	24 [18–44]	21 [17–28]	123 [63–231]	< 0.001
ALT/GPT (U/L)	21 [15–34.8]	20 [14–27]	39 [22–64]	< 0.001
White blood cells/µL	9250 [7340–11620]	8725 [7107–10870]	11,370 [9200–13720]	< 0.001
Neutrophils (%)	70.8 ± 32.8	67.10 ± 13.10	84.52 ± 65.12	< 0.001
Lymphocytes (%)	20.9 ± 11.1	22.61 ± 10.44	14.74 ± 11.71	< 0.001
Neutrophils/Lymphocytes ratio	3.5 [2.1–6.4]	3 [2–5.2]	6.4 [4–10.8]	< 0.001
D-Dimer (ng/mL) (*n* = 357)	453 [281–957]	420 [264–839]	742 [392–1749]	< 0.001
Blood glucose (mg/dL)	145.0 ± 67.5	141.20 ± 64.15	159.21 ± 77.76	0.021
High-sensitive troponin T (μg/L)	0.06 [0.017–0.405]	0.03 [0.01–0.14]	1.7 [0.4–4.2]	< 0.001
Creatine kinase MB (µg/L)	4.8 [2.4–24]	3.6 [2.1–9.1]	60.4 [15.2–172.6]	< 0.001
LDL (mg/dL)	97.8 ± 41.5	97.69 ± 39.50	98.06 ± 48.38	0.945
HDL (mg/dL)	42.7 ± 12.9	42.27 ± 13.08	44.45 ± 11.89	0.154
Triglycerides (mg/dL)	138.8 ± 72.2	141.02 ± 73.91	130.54 ± 65.08	0.225
Uric acid (mg/dL)	5.7 ± 2.6	5.77 ± 2.84	5.45 ± 1.85	0.388

*COPD* chronic obstructive pulmonary disease, *eGFR* glomerular filtration rate estimated with Modification of Diet in Renal Disease (MDRD) equation, *DAPT* dual antiplatelet therapy, *HDL* high-density lipoprotein, *LDL* low-density lipoprotein, *MI* myocardial infarction, *PAD* peripheral artery disease, *TIA* transient ischemic attack

Regarding pharmacological treatments at admission, patients with LF were less frequently on dual antiplatelet therapy (DAPT) (6% vs 13.9%, *p* = 0.060), especially P2Y12 inhibitor drugs (8.3% vs 19.3%, *p* = 0.021).

Patients with FIB-4 score > 3.25 had higher serum levels of WBCs (*p* < 0.001), neutrophils as % in WBC count (*p* < 0.001), CRP levels (*p* = 0.013), hs-TnT (*p* < 0.001), creatine-kinase MB (*p* < 0.001) and D-Dimer (*p* < 0.001). On the opposite, LF patients had lower percentage of lymphocytes (*p* < 0.001), platelets (*p* = 0.025), albumin levels (*p* = 0.018) on admission.

Supplementary Table 1 shows similar differences between patients with or without a high APRI score.

### ACS presentation and angiographic features of patients with or without LF

Presentation of ACS was STEMI in 44.3%, NSTEMI in 29% and UA in 26.7% (Table 2). UA was more common in non-LF patients (33% vs 3%, *p* < 0.001), whilst STEMI was more common in the LF group (71.7% vs 37%, *p* < 0.001). Patients with LF had more severe ACS presentation, with a higher Killip class (34.4% vs 13.3%, *p* < 0.001) and GRACE score upon admission (161.82 ± 40.92 vs 126.12 ± 36.55, *p* = 0.001; GRACE > 140 71.7% vs 63.5%, *p* < 0.001). No differences between the two groups were reported when analyzing angiographic data (Table 2).

**Table 2:** Clinical and angiography characteristics according to the FIB-4 score

	Overall (*n* = 469)	FIB-4 ≤ 3.25 (*n* = 305)	FIB-4 > 3.25 (*n* = 164)	*p*
Characteristics of ACS				
In-hospital stay (days)	10 [6–14]	9 [6–13]	13 [9–20]	< 0.001
ACS first episode (%)	319 (68.0)	245 (66.2)	74 (74.7)	0.116
Unstable angina (%)	125 (26.7)	122 (33.0)	3 (3.0)	< 0.001
NSTEMI (%)	136 (29.0)	111 (30.0)	25 (25.3)	0.385
STEMI (%)	208 (44.3)	137 (37.0)	71 (71.7)	< 0.001
Killip ≥ II (%)	79 (17.7)	47 (13.3)	32 (34.4)	< 0.001
Admission cardiogenic shock (%)	22 (4.7)	16 (4.3)	6 (6.1)	0.430
Admission cardiac arrest (%)	11 (2.4)	7 (1.9)	4 (4.1)	0.225
%LVEF at admission	44.3 ± 9.4	44.48 ± 9.28	43.52 ± 9.92	0.375
GRACE score	133.5 ± 40.2	126.12 ± 36.55	161.82 ± 40.92	< 0.001
Angiographic data				
Right dominance	392 (89.7)	313 (89.9)	79 (88.8)	0.700
LMCA ≥ 50% (%)	23 (5.2)	19 (5.4)	4 (4.5)	1.000
LAD ≥ 70% (%)	284 (64.1)	222 (62.7)	62 (69.7)	0.266
LCX ≥ 70% (%)	187 (42.2)	141 (39.8)	46 (51.7)	0.054
RCA ≥ 70% (%)	222 (50.2)	174 (49.3)	48 (53.9)	0.447
One-vessel disease (%)	173 (39.1)	137 (38.7)	36 (40.4)	0.808
Two-vessel disease (%)	124 (28)	98 (27.7)	26 (29.2)	0.792
Three or more vessel disease (%)	98 (22.1)	74 (20.9)	24 (27)	0.253
Culprit vessel in STEMI patients (*n* = 208)				
LMCA (%)	19 (9.1)	10 (7.3)	9 (12.7)	0.213
LAD (%)	45 (21.6)	26 (19)	19 (26.8)	0.216
LCX (%)	7 (3.4)	5 (3.6)	2 (2.8)	1.000
RCA (%)	36 (17.3)	27 (19.7)	9 (12.7)	0.248

*GRACE* Global Registry of Acute Coronary Events, *LAD* left anterior descending artery, *LCX* left circumflex artery, *LMCA* left main coronary artery, *LVEF* left ventricular ejection fraction, *NSTEMI* non-ST-elevation myocardial infarction, *RCA* right coronary artery, *STEMI* ST-elevation myocardial infarction

ACS and angiographic characteristics between patients with high or low APRI score were consistent with those obtained by using FIB-4 score (Supplementary Table 2).

### In-hospital adverse events

Overall, 71 patients experienced 110 AEs. The number and type of AEs according to presence or absence of a high FIB-4 score are reported in Table 3.

**Table 3:** Number and type of adverse events according to presence or absence of a high FIB-4 score

	Overall population (*n* = 469)	FIB-4 ≤ 3.25 (*n* = 370)	FIB-4 > 3.25 (*n* = 99)	*p*	Odds ratio (95% confidence interval)
Primary endpoints					
AEs (yes/no) (only the first AE) (%)	71 (15.1)	45 (12.2)	26 (26.3)	0.001	2.6 (1.5–4.4) *p* = 0.001
All AEs (%)	110 (23)	68 (18)	42 (42)	0.013	–
Cardiogenic shock (%)	44 (9.4)	27 (7.3)	17 (17.2)	0.006	2.6 (1.4–5.1) *p* = 0.004
PEA/asystole (%)	19 (4.1)	11 (3.0)	8 (8.1)	0.039	2.9 (1.12–7.34) *p = 0.028*
Acute pulmonary edema (%)	26 (5.5)	17 (4.6)	9 (9.1)	0.088	2.1 (0.9–4.8) *p* = 0.088
Cardiac death (%)	21 (4.5)	13 (3.5)	8 (8.1)	0.059	2.4 (0.97–6) *p* = 0.058
Other secondary endpoints					
Temporary pacing (%)	11 (2.4)	6 (1.6)	5 (5.1)	0.061	3.21 (0.9–10.7) *p* = 0.059
New onset atrial fibrillation (%)	46 (9.8)	34 (9.2)	12 (12.1)	0.446	1.4 (0.68–2.74) *p* = 0.385
2nd or 3rd degree atrioventricular block (%)	12 (2.6)	11 (3.0)	1 (1.0)	0.475	0.33 (0.04–2.6) *p* = 0.295
Non-sustained ventricular tachycardia (%)	82 (17.5)	56 (15.1)	26 (26.3)	0.016	2 (1.17–3.39) *p* = 0.011
Ventricular fibrillation (%)	26 (5.5)	20 (5.4)	6 (6.1)	0.806	1.13 (0.44–2.89) *p* = 0.800

*AEs* adverse events, *PEA* pulseless electrical activity

Table 4 reports clinical events occurring during the in-hospital staying according to the presence or absence of LF. Noteworthy, AEs were significantly more frequent in patients with LF than in those without (26.3% vs 12.2%, respectively, *p* = 0.001), with this difference being more evident in STEMI patients (Supplementary Fig. 1). In particular, cardiogenic shock (17.2% vs 7.3%, *p* = 0.006) and PEA/asystole (8.1% vs 3.0%, *p* = 0.039) were more frequent in LF patients, while cardiac deaths and acute pulmonary edema did not differ between the two groups.

**Table 4:** Characteristics of patients according to the composite endpoint of in-hospital adverse events

	Adverse events No (*n* = 398)	Adverse events Yes (*n* = 71)	*p*
Age (years)	65.2 12.6	68.8 14.7	0.032
Women (%)	91 (22.9)	17 (23.9)	0.879
Body mass index (kg/m^2^)	26.8 4.3	25.9 4.5	0.174
Liver Fibrosis Indexes			
FIB-4 score	2.38 ± 2.43	3.56 ± 3.38	< 0.001
FIB > 3.25 (%)	73 (18.3)	26 (36.6)	0.001
APRI score	0.73 ± 0.69	0.84 ± 0.62	0.232
APRI > 0.7 (%)	130 (32.7)	34 (47.9)	0.015
Characteristics of ACS			
In-hospital stay (days)	9 [6–14]	13 [9–19]	< 0.001
ACS first episode (%)	268 (67.3)	51 (71.8)	0.493
Unstable angina (%)	117 (29.4)	8 (11.3)	0.001
NSTEMI (%)	113 (28.4)	23 (32.4)	0.482
STEMI (%)	168 (42.2)	40 (56.3)	0.037
%LVEF at admission	46.02 ± 8.46	34.83 ± 9.47	< 0.001
GRACE score	128.94 ± 36.21	163.12 ± 54.11	< 0.001
Killip class ≥ II (%)	47 (12.4)	32 (46.4)	< 0.001
Admission cardiogenic shock (%)	10 (2.5)	12 (17.1)	< 0.001
Admission cardiac arrest (%)	7 (1.8)	4 (5.7)	0.067
One-vessel disease (%)	152 (40)	21 (33.3)	0.333
Two-vessel disease (%)	101 (26.6)	23 (36.5)	0.129
Three or more vessel disease (%)	80 (21.1)	18 (28.6)	0.192
History and risk factors			
Hypertension (%)	314 (78.9)	63 (88.7)	0.073
Diabetes (%)	115 (28.9)	27 (38)	0.125
COPD (%)	47 (11.8)	12 (16.9)	0.244
Smoking habit (%)	289 (73)	43 (62.3)	0.083
Current smoking (%)	165 (41.7)	22 (31.9)	0.144
PAD (upper + lower) (%)	20 (5)	2 (2.8)	0.554
Congestive heart failure history (%)	29 (7.3)	7 (10)	0.464
Thyroid disease (%)	38 (9.5)	4 (5.6)	0.370
History of stroke or TIA (%)	22 (5.5)	5 (7)	0.583
History of cancer (%)	51 (12.8)	7 (9.9)	0.562
Active cancer (%)	15 (3.8)	3 (4.2)	0.744
Prior MI (%)	114 (28.7)	16 (22.9)	0.386
Prior CABG (%)	33 (8.3)	4 (5.6)	0.632
Prior PCI (%)	105 (26.4)	13 (18.3)	0.182
Laboratory data			
Creatinine (mg/dL)	1.05 ± 0.67	1.36 ± 1.60	0.006
eGFR MDRD (mL/min)	83.8 ± 29.1	76.0 ± 37.6	0.051
eGFR < 60 mL/min (%)	71 (17.8)	22 (31.0)	0.015
Albumin (g/L) (*n* = 417)	39.79 ± 4.77	37.18 ± 5.02	< 0.001
Albumin < 36 g/L (%) (*n* = 417)	54/349 (15.5)	22/68 (32.4)	0.002
C-reactive protein (mg/dL) (*n* = 394)	0.6 [0.2–1.2]	1.3 [0.3–3.8]	< 0.001
Hemoglobin (g/dL)	14.49 ± 7.46	13.84 ± 2.00	0.467
Anemia (%)	74 (18.6)	19 (26.8)	0.145
Platelets (× 10^9^/L)	242.62 ± 81.9	245.77 ± 70.2	0.760
AST/GOT (U/L)	23 17–40.2	29 19–105	0.011
ALT/GPT (U/L)	21 [15–33]	24 [16–40]	0.075
White blood cells/µL	9000 [7235–11300]	10,530 [8320–14020]	< 0.001
Neutrophils (%)	70.0 ± 35.1	75.1 ± 13.59	0.228
Lymphocytes (%)	21.8 ± 10.89	16.13 ± 11.61	< 0.001
Neutrophils/lymphocytes ratio	3.2 [2.1–5.9]	6.4 [2.9–10.8]	< 0.001
D-Dimer (ng/mL) (*n* = 386)	436 [280–881]	751 [303–1786]	0.010
D-Dimer Peak (ng/mL) (*n* = 374)	595 [348–1212]	833 [430–4250]	0.005
Blood glucose (mg/dL)	141.63 ± 65.17	163.92 ± 75.52	0.012
High-sensitive troponin T (μg/L)	0.05 [0.02–0.27]	0.18 [0.02–2.24]	0.007
High-sensitive troponin T peak (μg/L)	0.74 [0.11–3.12]	2.02 [0.28–9.65]	0.001
Creatine kinase MB (µg/L)	4.3 [2.4–20.9]	6.3 [2.7–44.4]	0.129
Creatine kinase MB peak (µg/L)	17.8 [4.44–97.8]	44.5 [12.6–199]	0.002
LDL (mg/dL)	98.63 ± 41.8	93.44 ± 39.84	0.385
HDL (mg/dL)	42.7 ± 12.7	42.80 ± 13.75	0.966
Triglycerides (mg/dL)	137.68 ± 70.2	145.11 ± 82.88	0.452
Uric acid (mg/dL)	5.60 ± 2.68	6.19 ± 2.36	0.176

*ACS* acute coronary syndrome, *APRI* AST-to-platelet ratio index, *CABG* coronary artery bypass graft, *COPD* chronic obstructive pulmonary disease, *eGFR* glomerular filtration rate estimated with Modification of Diet in Renal Disease (MDRD) equation, *FIB-4* fibrosis 4, *GRACE* Global Registry of Acute Coronary Events, *HDL* high-density lipoprotein, *LDL* low-density lipoprotein, *LVEF* left ventricular ejection fraction, *MI* myocardial infarction, *NSTEMI* non-ST-elevation myocardial infarction, *PAD* peripheral artery disease, *PCI* percutaneous coronary intervention, *STEMI* ST-elevation myocardial infarction, *TIA* transient ischemic attack

Table 4 shows characteristics of patients experiencing or not AEs during hospitalization. Patients suffering from in-hospital AEs were characterized by an older age, longer in-hospital stay, lower values of LVEF, higher GRACE score and had more frequently STEMI and cardiogenic shock presentation. At laboratory analysis on admission, they also showed more frequently hypoalbuminemia and higher serum values of CRP, AST, WBC count, neutrophils/lymphocytes ratio, D-Dimer, glucose, hs-TnT and CK-MB (Table 4).

Table 5 shows univariable and multivariable odds ratio (OR) and 95% confidence interval (95% CI) of factors associated with in-hospital AEs.

**Table 5:** Univariable and multivariable logistic regression analysis of factors associated with in-hospital AEs (multivariable Model B including subgroup of patients with albumin C-reactive protein and D-Dimer *n* = 386)

	Univariable analysis		Multivariable analysis			
			Model A		Model B	
	OR (95% CI)	*p*	OR (95% CI)	*p*	OR (95% CI)	*p*
FIB-4 > 3.25	2.6 (1.5–4.4)	0.001	2.2 (1.1–4.2)	0.018	3.1 (1.4–6.9)	0.007
LVEF% on admission below median	6.2 (3.3–11.5)	< 0.001	6.9 (3.5–13.8)	< 0.001	9.2 (3.9–21.7)	< 0.001
Killip Class ≥ II	7.8 (3.6–17)	< 0.001	7.2 (2.9–17.5)	< 0.001	6.3 (2.2–18.4)	0.001
eGFR MDRD < 49 mL/min*	4.4 (2.4–8)	< 0.001	2.8 (1.4–5.6)	0.004	2.4 (1.02–5.5)	0.046
D-Dimer above median	1.8 (1.04–3.2)	0.036	–		0.6 (0.3–1.4)	0.229
Albumin below median	3.5 (2–6.2)	< 0.001	–		2.2 (1.03–4.7)	0.042
CRP above median	2.3 (1.3–4.1)	0.003	–		2.2 (1.02–5)	0.044

For the severity of acute coronary syndrome, we used first LVEF below the median and Killip Class ≥ 2

*CI* confidence interval, *CRP* C-reactive protein, *eGFR* glomerular filtration rate estimated with Modification of Diet in Renal Disease (MDRD) equation, *FIB-4* fibrosis 4 score, *LVEF* left ventricular ejection fraction, *OR* odds ratio, *ROC* receiver operating characteristic

*MDRD < 49 mL/min was obtained from ROC curve analysis

At the multivariable analysis, FIB-4 was found to be and independent predictor of poor outcomes (OR 3.1, 95%CI 1.4–6.9). Admission LVEF% below median was the more relevant independent predictor of in-hospital AEs (OR 9.2, 95%CI 3.9–21.7) followed by a Killip class ≥ II (OR 6.3, 95%CI 2.2–18.4) (Table 5). Moreover, eGFR MDRD < 49 ml/min (OR 2.4, 95%CI 1.02–5.5), albumin below median (OR 2.2, 95%CI 1.03–4.7) and CRP above median (OR 2.2, 95%CI 1.02–5) were also associated with in-hospital outcomes (Table 5).

When we repeated the analysis using the negative value of FIB-4 < 1.45 (rule out criterion for the presence of LF), we found an inverse association between low FIB-4 score and AEs at univariable OR: 0.45, 95%CI 0.24–0.86, *p* = 0.015, a marginally significant value at multivariable analysis (using Model A variables) with OR 0.55, 95%CI 0.30–1.01, *p* = 0.055).

## Discussion

The main findings of our prospective cohort study were that non-invasive diagnosis of LF was achieved in one out of five ACS patients, identifying patients with severe MI and at higher risk of in-hospital adverse events independent of traditional clinical and laboratory risk factors.

In our study on 469 patients with ACS, we found a 21.1% of LF prevalence as defined by a FIB-4 score > 3.25. In a study including 3263 patients with CAD in China, 1035 (31.7%) had a high FIB-4 defined by a value > 2.67 [14].

We found that patients with LF had a more severe ACS presentation. First, they suffered more frequently from hypoalbuminemia, thrombocytopenia and highest levels of inflammatory/severity markers such as hs-TnT, CRP, neutrophils to lymphocytes ratio and D-Dimer, all biomarkers associated with poor prognosis in ACS setting [15–19]. Then, in our population, MI clinical presentation was worse in LF patients. Indeed, they were more frequently admitted with STEMI and a higher Killip class and GRACE score. This is consistent with previous studies analyzing only STEMI patients who showed a very high percentage of LF/liver steatosis patients among them [11, 20].

We found a similar proportion of multivessel disease between patients with and without high FIB-4, that may be probably due to the high percentage of STEMI in the former group.

Interestingly, LF patients were less frequently on DAPT therapy, especially P2Y12 inhibitors, on admission. The association between antiplatelet therapy and LF has been investigated in the past and a protective association between the use of antiplatelet agents and the occurrence of liver fibrosis has been demonstrated in a prospective cohort study of patients at high risk of cardiovascular events [21]. Previous data have shown a pathogenic role of platelets in the pathogenesis of LF, and antiplatelet agents was found to be effective in attenuating liver steatosis in preclinical studies [22, 23].

We also investigated the association between LF and in-hospital outcomes. We found that LF as detected by an easy inexpensive non-invasive score calculated with routine laboratory data was independently associated with in-hospital AEs independently from traditional clinical and laboratory risk factors. The association between LF and mortality has been investigated only in one previous study performed in China using a lower FIB-4 cut-off, in which Chen et al. found that higher LF scores were associated with increased risks of all-cause and cardiovascular mortality among CAD patients followed for 7.5 years [14].

The pathophysiology linking between LF and CAD is unclear but may rely on different mechanisms. LF and eventually liver steatosis have been associated with increased levels of pro-inflammatory cytokines, such as tumor necrosis factor α, interleukin 6, and CRP, biomarkers well known to have an impact on CAD and cardiovascular prognosis [24]. As well, advanced LF has been shown to be associated with endothelial dysfunction increasing atherosclerosis [25]. Previous studies have shown an association between LF assessed with non-invasive markers and imaging indicators of atherosclerosis such as carotid mean intimal thickness and coronary calcium score [26, 27].

Novel pathogenetic insights come from the study of gut and gut-derived products. Thus, an increased liver localization of gut-derived lipopolysaccharides (LPS) has been found in patients with liver disease, associated with increased liver inflammation [28].

Additionally, a growing body of evidence suggest that LPS may contribute to increase cardiovascular risk favoring platelet activation [29, 30] and that LPS may found into human atherosclerotic plaque potentially contributing to plaque vulnerability and in turn favoring plaque rupture [31, 32]. Indeed, in ACS patients with LF, LPS may contribute to develop a more severe coronary disease. This aspect deserves further study.

Our study had some limitations. First, this was a single-center study and results must be confirmed in larger studies. Although liver biopsy is the best standard to confirm/exclude LF, it is invasive, expensive, prone to sampling error, and can cause rare but potentially life-threatening complications. Nowadays, it has been almost entirely replaced by non-invasive methods that measure liver stiffness, such as transient elastography, or biochemical markers and scoring systems. In terms of cost-effectiveness, considering the high prevalence of LF and CAD, biochemical markers, such as the APRI score and the FIB-4 score, recently shown to be reliable in predicting LF and validated in NAFLD population [13, 33], may be helpful to identify ACS patients at higher risk of complications and events. Furthermore, in our analysis a limited number of patients had a known liver disease (viral or/and alcoholic) that does not allow a specific analysis on the subtypes of LF. Further studies are needed to investigate the impact of different liver conditions on ACS patients.

In conclusion, LF was associated with a more severe presentation and worse in-hospital AEs in ACS patients undergoing PCI. Our findings suggest that the detection of LF with non-invasive and inexpensive scores may represent a simple method to improve risk stratification and management of patients with ACS.

## Supplementary Information

Below is the link to the electronic supplementary material.

Supplementary file1 (DOCX 120 KB)

## Abbreviations

ACS: Acute coronary syndrome
AEs: Adverse events
AF: Atrial fibrillation
ALT: Alanine aminotransferase
APRI: AST-to-platelet ratio index
AST: Aspartate aminotransferase
CAD: Coronary artery disease
CI: Confidence interval
CRP: C-reactive protein
DAPT: Dual antiplatelet therapy
eGFR: Estimated glomerular filtration rates
FIB-4: Fibrosis 4
GRACE: Global Registry of Acute Coronary Events
hs-TnT: High-sensitive troponin T
LVEF: Left ventricular ejection fraction
LF: Liver fibrosis
MI: Myocardial infarction
NSTEMI: Non-ST-elevation myocardial infarction
OR: Odds ratio
PCI: Percutaneous coronary intervention
PEA: Pulseless electrical activity
ROC: Receiver operating characteristic
STEMI: ST-elevation myocardial infarction
UA: Unstable angina
WBCs: White blood cells
