# Supplementary material for: Relationship between non-invasively detected liver fibrosis and in-hospital outcomes in patients with acute coronary syndrome undergoing PCI
Source: Clin Res Cardiol. 2022 Aug 11;112(2):236–46. doi: 10.1007/s00392-022-02078-z (PMC9898425; doi:10.1007/s00392-022-02078-z)
Supplement: Supplementary file 1 — Supplementary file1 (DOCX 120 KB) [file 392_2022_2078_MOESM1_ESM.docx]

**SUPPLEMENTARY DATA**

**Supplementary Figure 1. Rates of adverse events according to presence or absence of liver fibrosis (FIB-4 score >3.25) in patients with ST-elevation myocardial infarction, non-ST elevation myocardial infarction and unstable angina.**

**
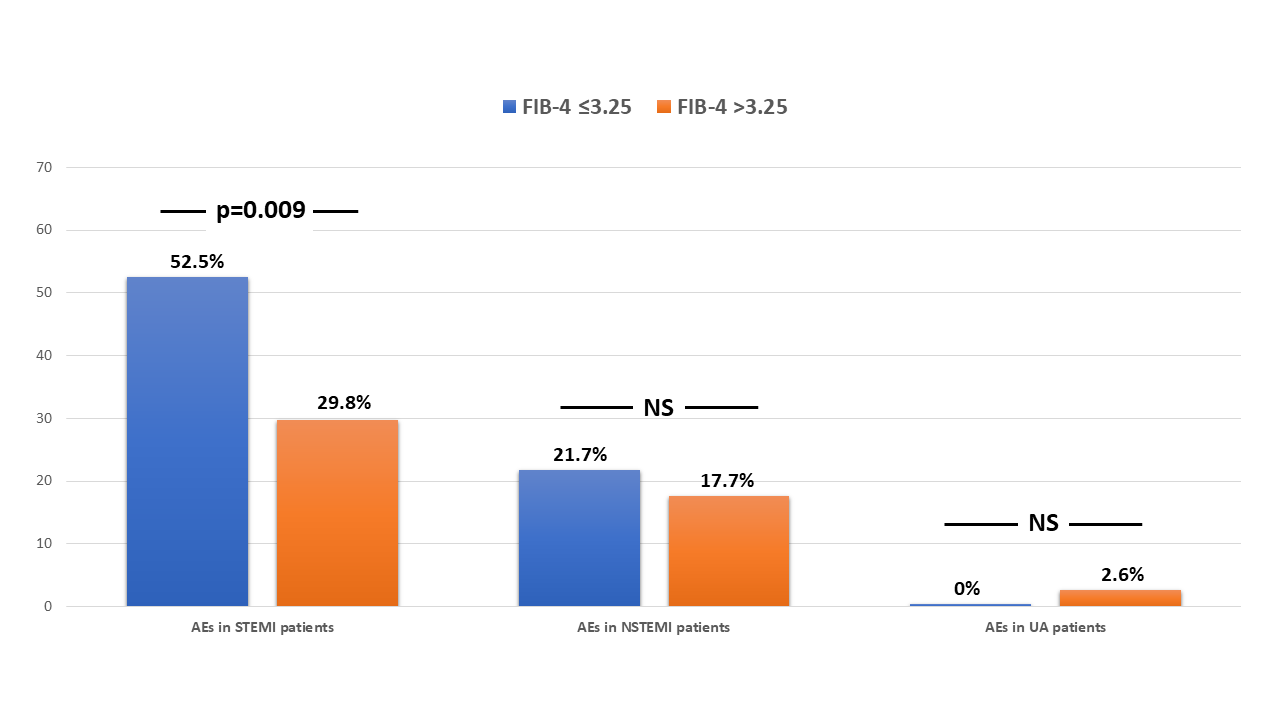
**

**Supplementary Table 1. Characteristics of patients according to APRI values.**

|  | Overall  (n=469) | APRI ≤0.7  (n=305) | APRI >0.7  (n=164) | p |
| --- | --- | --- | --- | --- |
| Age (years) | **65.7±13.0** | **64.4±12.4** | **68.1±13.8** | **0.004** |
| Women (%) | 108 (23.0) | 63 (20.7) | 45 (27.4) | 0.108 |
| Body mass index (Kg/m^2^) | **26.7±4.3** | **27.1±4.2** | **25.9±4.4** | **0.006** |
| Risk factors | | | | |
| Hypertension (%) | 377 (80.4) | 252 (82.6) | 125 (76.2) | 0.113 |
| Diabetes (%) | 142 (30.3) | 98 (32.1) | 44 (26.8) | 0.248 |
| COPD (%) | 59 (12.6) | 35 (11.5) | 24 (14.6) | 0.381 |
| Smoking habit (%) | 332 (71.4) | 222 (73.5) | 110 (67.5) | 0.197 |
| Current Smoking (%) | 187 (40.2) | 122 (40.4) | 65 (39.9) | 0.921 |
| PAD (upper + lower) (%) | 95 (20.3) | 60 (19.7) | 35 (21.3) | 0.718 |
| Congestive heart failure history (%) | 36 (7.7) | 27 (8.9) | 9 (5.5) | 0.208 |
| Permanent atrial fibrillation (%) | 18 (3.8) | 8 (2.6) | 10 (6.1) | 0.078 |
| Thyroid Disease (%) | 42 (9.0) | 31 (10.2) | 11 (6.7) | 0.239 |
| History of stroke or TIA (%) | 27 (5.8) | 18 (5.9) | 9 (5.5) | 1.000 |
| History of cancer (%) | 58 (12.4) | 33 (10.8) | 25 (15.2) | 0.186 |
| Prior MI (%) | 130 (27.8) | 92 (30.3) | 38 (23.3) | 0.129 |
| Home therapy | | | | |
| ACE inhibitors (%) | 241 (53.5) | 89 (34.8) | 39 (27.1) | 0.119 |
| Aldosterone receptor antagonists (%) | 11 (2.8) | 52 (20.3) | 36 (25.0) | 0.315 |
| Beta blockers (%) | 139 (34.8) | 95 (37.1) | 44 (30.6) | 0.192 |
| Acetylsalicylic acid (%) | **167 (41.8)** | **120** (**46.9**) | **47** (**32.6**) | **0.006** |
| P2Y12 inhibitors (%) | **68 (17.0)** | **51** (**19.9**) | **17** (**11.8**) | **0.039** |
| DAPT (%) | **49 (12.3)** | **40 (15.6)** | **9 (6.3)** | **0.006** |
| Acetylsalicylic acid or P2Y12 inhibitors (%) | **186 (46.5)** | **131 (51.2)** | **55 (38.2)** | **0.016** |
| Proton pump inhibitors (%) | 138 (34.5) | 92 (35.9) | 46 (31.9) | 0.445 |
| Oral anticoagulants (any) (%) | 28 (7.0) | 20 (7.8) | 8 (5.6) | 0.541 |
| Non-dihydropyridine calcium channel blockers (%) | 9 (2.3) | 7 (2.7) | 2 (1.4) | 0.498 |
| Dihydropyridine calcium channel blockers (%) | 106 (26.5) | 67 (26.2) | 39 (27.1) | 0.906 |
| Ranolazine (%) | 23 (5.8) | 15 (5.9) | 8 (5.6) | 1.000 |
| Statins (%) | **124 (31.0)** | **89** (**34.8**) | **35** (**24.3**) | **0.033** |
| Cholesterol-absorption inhibitors (%) | 9 (2.3) | 6 (2.3) | 3 (2.1) | 1.000 |
| Statins/cholesterol-absorption inhibitors association (%) | 9 (2.3) | 7 (2.7) | 2 (1.4) | 0.498 |
| Fibrates (%) | 3 (0.8) | 3 (1.2) | 0 (0.0) | 0.556 |
| Oral antidiabetic drugs (%) | 76 (19.0) | 51 (19.9) | 25 (17.4) | 0.596 |
| Insulin therapy (%) | 28 (7.0) | 17 (6.6) | 11 (7.6) | 0.689 |
| Uric acid lowering therapy (%) | 19 (4.8) | 12 (4.7) | 7 (4.9) | 1.000 |
| Laboratory data | | | | |
| Creatinine (mg/dl) | 1.09±0.88 | 1.1±0.9 | 1.1±0.8 | 0.816 |
| Albumin (g/L) (n=417) | **39.4±4.8** | **39.9±4.7** | **38.4±4.9** | **0.003** |
| Albumin <36 g/L (%) (n=417) | **76 (18.2)** | **41 (15.1)** | **35 (24.1)** | **0.024** |
| C-reactive protein (mg/dl) (n=356) | **0.6[0.2-1.6]** | **0.6 [0.2-1.2]** | **0.9 0.3 2.1** | **0.017** |
| Haemoglobin (g/dl) | 14.4±6.9 | 14.6**±**8.5 | 14.0**±**1.9 | 0.342 |
| Anaemia (%) | 93 (19.8) | 57 (18.7) | 36 (22.0) | 0.398 |
| Platelets (x10^9^/µL) | **243.1±80.2** | **257.1±84.6** | **217.1±63.8** | **<0.001** |
| Thrombocytopenia <150 x10^9^/µL (%) | **26 (5.5)** | **7 (2.3)** | **19 (11.6)** | **<0.001** |
| AST/GOT (U/l) | **24 [18-44]** | **21 [17-28** | **46 [21.2-126.7]** | **<0.001** |
| ALT/GPT (U/l) | 21 [15-34.8] | 21 [17-32.5] | 20.5 [12-39] | 0.092 |
| White Blood Cells/µL | **9250 [7340-11620]** | **8740 [7155-10855]** | **10240 [8045-12702]** | **<0.001** |
| Neutrophils (%) | **70.8±32.8** | **66.8±13.5** | **78.2±51.6** | **<0.001** |
| Lymphocytes (%) | **20.9±11.1** | **22.8±10.6** | **17.6±11.5** | **<0.001** |
| Neutrophils/Lymphocytes ratio | **3.5 [2.1-6.4]** | **3 [1.9-5.1]** | **5.3 [2.9-8.7]** | **<0.001** |
| D-Dimer (ng/ml) (n=357) | **453 [281-957]** | **421 [262.5-838]** | **594 [325-1248]** | **<0.001** |
| Blood glucose (mg/dl) | 145.0±67.5 | 143.7±68 | 147.4±66.7 | 0.577 |
| High-sensitive troponin T (μg/L) | **0.06 [0.017-0.405]** | **0.03 [0.01-0.11]** | **0.3 [0.05-2.5]** | **<0.001** |
| Creatine kinase MB (µg/L) | **4.8 [2.4-24]** | **3.4 [2.1-8.1]** | **20.9 [4.2-100.4]** | **<0.001** |
| LDL (mg/dl) | 97.8±41.5 | 98.6±40.4 | 96±43.8 | 0.573 |
| HDL (mg/dl) | 42.7±12.9 | 41.9±12.8 | 44.4±12.9 | 0.052 |
| Triglycerides (mg/dl) | 138.8±72.2 | 142.6±68.5 | 131.5±78.5 | 0.135 |
| Uric Acid (mg/dL) | 5.7±2.6 | 5.6±2.7 | 5.8±2.5 | 0.531 |

**Supplementary Table 2. Clinical and angiography characteristics according to APRI score.**

|  | Overall  (n=469) | | APRI ≤0.7  (n=305) | | APRI >0.7  (n=164) | p |
| --- | --- | --- | --- | --- | --- | --- |
| Characteristics of ACS | | | | | | |
| In-hospital stay (days) | | **10 [6-14]** | | **8 [6-13]** | **12 [8-16]** | **<0.001** |
| ACS first episode (%) | | 319 (68.0) | | 197 (64.6) | 122 (74.4) | 0.038 |
| Unstable angina (%) | | **125 (26.7)** | | **102 (33.4)** | **23 (14)** | **<0.001** |
| NSTEMI (%) | | 136 (29.0) | | 92 (30.2) | 44 (26.8) | 0.458 |
| STEMI (%) | | **208 (44.3)** | | **111 (36.4)** | **97 (59.1)** | **<0.001** |
| Killip ≥II (%) | | **79 (17.7)** | | **38 (13)** | **41 (26.6)** | **0.001** |
| Admission Cardiogenic Shock (%) | | 22 (4.7) | | 14 (4.6) | 8 (5.0) | 0.823 |
| Admission Cardiac Arrest (%) | | 11 (2.4) | | 6 (2.0) | 5 (3.1) | 0.524 |
| %LVEF at admission | | 44.3±9.4 | | 44.1**±9.2** | 44.5**±**9.7 | 0.656 |
| GRACE score | | **133.5±40.2** | | **125.6±39.1** | **147.7±38.1** | **<0.001** |
| Angiographic Data | | | | | | |
| Right Dominance | | 392 (89.7) | | 255 (90.4) | 137 (88.4) | 0.514 |
| LMCA ≥50% (%) | | 23 (5.2) | | 13 (4.5) | 10 (6.5) | 0.376 |
| LAD ≥70% (%) | | 284 (64.1) | | 177 (61.5) | 107 (69) | 0.120 |
| LCX ≥70% (%) | | 187 (42.2) | | 115 (39.9) | 72 (46.5) | 0.191 |
| RCA ≥70% (%) | | 222 (50.2) | | 139 (48.3) | 83 (53.5) | 0.319 |
| One-vessel disease (%) | | 173 (39.1) | | 110 (38.2) | 63 (40.6) | 0.683 |
| Two-vessel disease (%) | | 124 (28) | | 83 (28.8) | 41 (26.5) | 0.658 |
| Three or more vessel disease (%) | | 98 (22.1) | | 56 (19.4) | 42 (27.1) | 0.072 |
